# Health-related quality of life in the Cambridge City over-75s Cohort (CC75C): development of a dementia-specific scale and descriptive analyses

**DOI:** 10.1186/1471-2318-14-18

**Published:** 2014-02-10

**Authors:** Jaime Perales, Theodore D Cosco, Blossom CM Stephan, Jane Fleming, Steven Martin, Josep Maria Haro, Carol Brayne

**Affiliations:** 1 Parc Sanitari Sant Joan de Déu, Sant Boi de Llobregat, Barcelona, Spain; 2 Department of Public Health & Primary Care, Cambridge Institute of Public Health, University of Cambridge, Cambridge, UK; 3 The Institute of Health and Society, Newcastle University, Newcastle upon Tyne, UK; 4 Instituto de Salud Carlos III, Centro de Investigación Biomédica en Red de Salud Mental, CIBERSAM, Madrid, Spain

**Keywords:** Health-related quality of life, Dementia, Alzheimer’s disease

## Abstract

**Background:**

The assessment of Health Related Quality of Life (HRQL) is important in people with dementia as it could influence their care and support plan. Many studies on dementia do not specifically set out to measure dementia-specific HRQL but do include related items. The aim of this study is to explore the distribution of HRQL by functional and socio-demographic variables in a population-based setting.

**Methods:**

Domains of DEMQOL’s conceptual framework were mapped in the Cambridge City over 75’s Cohort (CC75C) Study. HRQL was estimated in 110 participants aged 80+ years with a confirmed diagnosis of dementia with mild/moderate severity. Acceptability (missing values and normality of the total score), internal consistency (Cronbach’s alpha), convergent, discriminant and known group differences validity (Spearman correlations, Wilcoxon Mann-Whitney and Kruskal-Wallis tests) were assessed. The distribution of HRQL by socio-demographic and functional descriptors was explored.

**Results:**

The HRQL score ranged from 0 to 16 and showed an internal consistency Alpha of 0.74. Validity of the instrument was found to be acceptable. Men had higher HRQL than women. Marital status had a greater effect on HRQL for men than it did for women. The HRQL of those with good self-reported health was higher than those with fair/poor self-reported health. HRQL was not associated with dementia severity.

**Conclusions:**

To our knowledge this is the first study to examine the distribution of dementia-specific HRQL in a population sample of the very old. We have mapped an existing conceptual framework of dementia specific HRQL onto an existing study and demonstrated the feasibility of this approach. Findings in this study suggest that whereas there is big emphasis in dementia severity, characteristics such as gender should be taken into account when assessing and implementing programmes to improve HRQL.

## Background

Dementia is the most common disorder of old age [1,2] and a leading cause of mortality and disability in high income countries [3]. Medications temporarily relieve symptoms for some individuals, but do not modify the overall course of the disease, [4]. Narrow assessment of cognition and functional ability is insufficient for clinical decision-making and policy development as they only reflect a part of the impact of dementia [5]. Dementia can also have a significant impact on quality of live (QoL). Indeed, treatment is increasingly focused on improving or maintaining optimal QoL and this has become a key outcome for evaluating the effectiveness of dementia interventions [5-7].

Health-related quality of life (HRQL) can be defined as an individual’s perception of the impact of a health condition on their everyday life [8]. HRQL differs from the broader concept of QoL in that it includes only aspects of QoL that are affected by a health condition. Despite the lack of agreement on what domains constitute HRQL, it is generally agreed that the concept is multidimensional and subjective and that any assessment should include measurement of positive and negative dimensions [9-12]. There have been many efforts to capture HRQL in dementia including generic and dementia-specific HRQL measures. Generic HRQL measures are not tailored to people with dementia [13,14]. Such measures focus on health and function and imply that HRQL will automatically decrease with disease progression. Some generic measures (e.g., Nottingham Health Profile [15], Duke Health Profile [16]) have been found to lack evidence on validity or reliability in dementia populations [17]. A recent recent systematic review on HRQL in dementia found that there are at least 15 different dementia specific HRQL scales [18]. Some of the most widely used include: the Quality of Life in Alzheimer’s Disease (QoL-AD) [19], Alzheimer’s Disease Related Quality of Life (ADRQL) [13], QUALIDEM [20], Quality Of Life Assessment Schedule (QOLAS) [21] and the DEMQOL [22]. Each of these measures a wide range of dimensions and their use has been found to be beneficial within a clinical framework. However, there are limitations with each. For example, while the QoL-AD has been validated in numerous countries it includes items on functioning and cognition implying that HRQL will decrease automatically with disease progression. The ADRQL can obtain both total and subscale scores. However it is a proxy-rated only measure and therefore is not the most suitable measure for use in individuals with mild-moderate dementia. The same applies to the QUALIDEM. In the QOLAS scale, while the subdomains are chosen by the patient, making this one of the most suitable measures for clinical practice, this limits its utility for research purposes since the concept of HRQL is intrinsic to the person. The DEQMOL’s conceptual framework was developed by literature review and interviews with people with dementia and their carers. There is a self and a proxy-rated version. However, in a clinical sample, factor analysis showed a lack of dimensionality. Lastly, a limitation of all scales is that none has been validated in a population-based sample.

Although research on HRQL has increased in the last decade, there is still limited evidence on how it is affected by dementia [23]. Knowledge of this is necessary to measure the impact of early diagnosis, access to treatment and effective planning of care. To our knowledge no study has assessed HRQL with dementia-specific measures in a population-based setting. While many population studies focused on dementia do not specifically set out to measure dementia-specific HRQL, they do include related items such as mood or social support. Mapping measures of HRQL in such studies is important for determining the distribution of HRQL and its risk factors in the dementia population. Therefore, the aim of this study was to explore the distribution of HRQL by functional and socio-demographic variables, mapped using items of the DEMQOL scale, using data from the Cambridge City over 75s Cohort (CC75C).

## Methods

### Sample and design

Participants were from the CC75C study, a population-based longitudinal cohort study representative of Cambridge’s (UK) older people. Full details of the methodology has been described elsewhere [24] and can be found online at can be found on line at http://www.cc75c.group.cam.ac.uk/. The baseline survey enrolled 95% (n = 2610) of individuals aged 75 years and older, approached from six general practices in Cambridge in 1985/87. Participants were followed with surveys repeated at two (Survey 2), seven (Survey 3), ten (Survey 4), 13 (Survey 5), 17 (Survey 6) and 21 years (Survey 7). Interviews were conducted with the study participants and a proxy informant if the participant was unable to complete the interview. Proxy informants were usually relatives but could sometimes be a friend/neighbour or a warden or member of staff in a care home. Observations by the interviewer were also gathered during the course of the interviews. Core data included information on socio-demographic variables (e.g. place of residence, household structure, marital status and social contact), activities of daily living (ADLs), use of health and social services, cognitive function (e.g., Mini Mental State Examination (MMSE) [25]), health problems and medication. This data except for HRQL and cognitive assessments was retrieved from the caregivers if necessary. The interview schedule has undergone slight revisions with the addition of new sections, such as questions on affect and loneliness added in survey 3 and 4. Survey 3, conducted in the 7^th^ year of follow up, was the first survey that best represented all the DEMQOL domains, with questions including socio-demographic information, social relationships, mood, loneliness, anxiety, depression, ADLs and cognitive functioning. Therefore data from Survey 3 will be used in this analysis.

### Dementia diagnosis

Dementia was diagnosed using a two-stage approach. At baseline, individuals who scored ≤ 23 on the MMSE, and one in three individuals with a MMSE score of 24 or 25, were assessed using the Cambridge Mental Disorders of the Elderly Examination (CAMDEX) [26], a structured schedule specifically designed to detect mild dementia. Between the baseline interview and survey 3, six assessments for dementia were conducted. Based on the CAMDEX results, different dementia types were assessed including: Alzheimer’s disease (AD), vascular dementia (VaD), mixed (AD and VaD), dementia secondary to other causes, clouded/delirious state plus AD and clouded/delirious state plus VaD. Dementia diagnoses were also sought by checking death certificates for any mention of dementia as a cause of death and checking reports from interviews with relatives for the sub-sample of participants who donated their brains to research. No additional people with dementia were identified. For the present study, participants with a last diagnosis of dementia, from any CAMDEX assessment and MMSE score > 10 (n = 110, 40 men and 70 women aged 80-99) up until one year after Survey 3 were identified for inclusion in this analysis. Among those without a MMSE score, 2 people were diagnosed with minimal, 2 with mild and 2 with moderate dementia using the CAMDEX. The staging of dementia was calculated using cut-offs on the MMSE as follows: 11–20 = moderate; 21 and above = mild [27].

### Assessment of socio-demographic and functional ability

Socio-demographic variables included age (continuous), gender, marital status (currently married, widowed, separated/divorced, and never married), education (up to 14 years of age and 15 and above) and accommodation (house, warden controlled housing, residential home and long stay hospital). Functional disability included impairment in basic and instrumental ADLs (IADLs). Individuals were categorised into three groups based on their pattern of impairment including: (1) IADL disability only if they required help with cooking, housework or both; (2) ADL + IADL disability if they needed help with any of four basic ADL tasks including: bathing, dressing, getting to the toilet on time and grooming; or, (3) No ADL/IADL disability.

### Questionnaire mapping and development

In this study we chose to map the DEMQOL framework as it includes the domains that have been most commonly assessed in dementia-specific HRQL measures, namely daily activities/looking after yourself, health and well-being, cognitive functioning, social relationships, and self-concept. Further, the conceptual framework underpinning the DEMQOL was developed in in the UK. Using data obtained at Survey 3, the following subdomains of the DEMQOL framework could be mapped including: health and well-being, social relationships, self-concept and items from daily activities corresponding to enjoyment of activities. Only self-reported information was used to assess HRQL. Due to the high percentage of missing responses to HRQL items in the most cognitively impaired participants, only those with a MMSE score above 10 were included in the study, as has been suggested previously [28-30].

Table 1 shows the DEMQOL conceptual framework and the subdomains that could be mapped in the CC75C study. After mapping, 18 items were included. After applying existing criteria for missing data analysis, inter-item correlations and endorsement frequencies analysis [22], two of these items were removed, giving a scale ranging from 0 to 16 with 0 being the lowest and 16 the highest scores.

**Table 1:** DEMQOL conceptual framework and mappin

**DEMQOL domain**	**Item in the CC75C study**
Daily activities^1^	
-getting around	NA
-keeping yourself clean	NA
-keeping yourself looking nice	NA
-going to the toilet	NA
-using knife and fork	NA
-getting the things you need from the shops	NA
-getting in touch with people when you need to	NA
-getting meals	NA
-taking care of the house	NA
-getting where you need to go	NA
-taking care of your finances	NA
-using money to buy everyday things	NA
-choice about how to spend your time	No
-things that you want to do but you can’t	No
-being able to enjoy what you do	The things I do are as interesting to me as they ever were
	Have you lost pleasure or interest in doing things you usually cared about or enjoyed?
Cognitive functioning^1^	
-memory for recent events	NA
-memory for names	NA
-concentration	NA
-orientation in time, place and person	NA
-clarity of thought	NA
-making your mind up	NA
-communication	NA
Self-concept	
-self-esteem	No
-presentation of self	No
-sense of independence	No
-satisfaction with past life	No
-satisfaction with present life	No
-hopes and aspirations for the future	How do you feel about the future?
-feeling useful	No
Health and well-being	
-global health rating	How would you rate your physical health compared to *a year ago*?
-happiness-depression	I am just as happy as when I was younger
-contentment-frustration	No
-enjoying life-enjoying nothing	Would you say that you enjoy your life?
-confidence	No
-embarrassment	No
-anxiety	Do you feel more tense and worry more than usual about little things?
-lively-weary	Would you say you have more or less energy at the moment than you did *a year ago?*
-loneliness	Do you feel lonely?
-somatic complaints	Have you had more trouble sleeping recently than is normal for you?
	Have you lost *(or gained)* a lot of weight in the last six months?
-feeling safe	No
-cheerful	No
-relaxed	No
-irritable	Have you felt more irritable lately?
-angry	No
-resentful	No
-sad	Do you feel sad or depressed or miserable?
-distressed	No
Social relationships	
-treatment by others	No
-social interaction	No
-reciprocity	Is there anyone whom you help with anything?
-social integration	No
-companionship	No
-social support	There are members of my family (friends) who can be relied on no matter what happens
-intimacy and physical affection	In general, do you have as much contact with family and friends as you would like to?
-other emotional relationships	No

^1^Cognition and other daily activities were excluded since they are symptoms of dementia.

### Analysis

The psychometric properties of the final version of the questionnaire (16 items) were tested with the sample of 110 people with dementia identified as described above. Standard psychometric methods were used [31] to evaluate acceptability, reliability (internal consistency) and validity (content, convergent, discriminant and known group differences). Criteria for acceptability were: missing data for summary scores <5% and normality of the distribution of the total score (Skewness measured with Shapiro-Francia, Shapiro-Wilk and Skewness test with a p value higher than 0.05 indicating normality). Criteria for internal consistency were Cronbach’s alpha ≥0.7. Convergent and discriminant validity were measured with Spearman correlations (r). Convergent validity is the evidence that the scale is correlated with other measures of the same or similar constructs and was deemed acceptable when r was 0.20 and higher. Discriminant validity is the evidence that the scale is not correlated with measures of different constructs and was deemed acceptable when r was below 0.20. Known group differences refer to the ability of a scale to differentiate groups who are expected to differ on the construct being measured. Differences in HRQL scores by socio-demographic, functional and measures of disability and cognition were tested using the Wilcoxon Mann-Whitney tests (variables with two groups) and Kruskal-Wallis test (variables with more than two groups). Statistical significance was set at p < 0.05. A meta-analytical review was conducted in order to generate *a priori* hypotheses as part as the validity assessment of the measure used. This has been annexed as a separate report (Additional file 1) as a separate report. We used factor analysis to evaluate hypothesised subscales based on the conceptual framework. All analyses were performed using Stata 11 [32].

### Ethics approval and consent

Each CC75C study phase was approved by Cambridge Research Ethics Committee and National Research Ethics Service Committee East of England- Cambridge Central (Reference numbers: 05_Q0108_308 and 08_H0308_3).

Written informed consent was obtained from all participants. For those deemed to lack capacity to give fully informed consent, and those unable to give consent in writing for reasons of physical disability, both the study participant’s clear assent to be interviewed and written proxy informed consent from a relative or caregiver well known to the participant were attained.

## Results

### Description of sample

A description of the sample can be found in Table 2. Out of the 713 people who took part in Survey 3, there were 134 people with a dementia diagnosis. From these, we excluded n = 24 with MMSE scores 0-10. Diagnoses included Alzheimer’s disease (69.1%), vascular dementia (3.6%), mixed dementia (22.7%) and other dementia (4.6%). Age ranged from 81 to 99 years (mean age 86.6, SD 3.6), 63.6% were women and 29.1% of the sample left school at age 15 or later. A high percentage of the sample lived in a house or flat (70.9%), 16.4% lived in warden controlled sheltered housing and 11.8% in a residential home. Most participants were widowed (77.1%), with less than a quarter of the sample (23.6%) married. In terms of disability 31.8% had no ADL/IADL disability, 30.9% had only IADL disability and 36.4% had both. A higher percentage of women with dementia had both ADL and IADL disability compared to men (44.3% vs. 22.5%). Scores on the MMSE scores ranged from 11 to 30 (mean MMSE 21.3, SD 4.8). Dementia severity was rated mild for 60% of the sample and 40% had moderate dementia. A high percentage of patients (78.2%) rated their health as good or very good.

**Table 2:** Socio-demographic profile of the sample

**Sociodemographic and health status variables by sex**	**Men (n = 40)**		**Women (n = 70)**		**Total (n = 110)**	
	**n**	**%**	**n**	**%**	**n**	**%**
Age						
Mean (SD)	86.6	4.0	86.2	3.4	86.6	3.6
Range	81	96	81	96	81	96
81–84	19	47.5	30	42.9	49	44.5
85–89	14	35.0	32	45.7	46	41.5
90+	7	17.5	8	11.4	15	13.6
Education						
Left school <15 years old	29	72.5	49	70.0	78	70.9
Left school ≥15 years old	11	27.5	21	30.0	32	29.1
Accommodation						
House/flat/granny flat	28	70.0	50	71.4	78	70.9
Warden controlled/sheltered housing	7	17.5	11	15.7	18	16.4
Residential home	4	10.0	9	12.9	13	11.8
Long stay hospital	1	2.5	0	0.0	1	0.9
Marital status						
Married	16	40.0	10	14.3	26	23.6
Widowed	20	50.0	54	77.1	74	67.3
Separated/divorced	0	0.0	1	1.4	1	0.9
Never married	4	10.0	5	7.1	9	8.2
Disability						
No ADL or IADL	13	32.5	22	31.4	35	31.8
IADL only	18	45.0	16	22.9	34	30.9
ADL + IADL	9	22.5	31	44.3	40	36.4
Missing	0	0.0	1	1.4	1	0.9
MMSE						
Mean (SD)	22.0	4.7	20.9	4.8	21.3	4.8
Range	13	30	11	29	11	30
Moderate	14	35.0	30	42.9	44	40.0
Mild	26	65.0	40	57.1	66	60.0
Self-rated health						
Very good/good	34	85.0	52	74.3	86	78.2
Fair/very poor	4	10.0	10	14.3	14	12.7
Missing	2	4.7	8	10.8	10	8.6
Dementia type						
Alzheimer’s Disease (AD)	30	75.0	46	65.7	76	69.9
Vascular Dementia (VD)	1	2.5	3	4.3	4	3.6
Mixed (AD + VD)	7	17.5	18	25.7	25	22.7
Dementia secondary to other causes	1	2.5	1	1.4	2	1.8
Clouded/delirious state + AD	0	0.0	2	2.9	2	1.8
Clouded/delirious state + VD	1	2.5	0	0.0	1	0.9

### Results from mapping

We were able to map all the DEMQOL domains except for those that are dementia symptoms (cognition and ADLs except for enjoyment of activities). One out of seven subdomains of self-esteem, nine out of eighteen of health and well-being, three out of eight of social relationships and one out of fifteen of daily activities (one out of four of enjoyment of activities) were mapped onto the CC75C study.

### Psychometric evaluation

Table 3 shows the psychometric properties of the HRQL scale assessed [31]. Regarding acceptability, missing data was below the criteria of 5% (2.73%). Shapiro-Francia, Shapiro-Wilk and Skewness test indicated that the distribution of HRQL was skewed. The HRQL questionnaire shows acceptable internal consistency reliability (α = 0.73). Regarding convergent validity, correlations with cognition measured with the MMSE as a continuous variable and disability were not significant (r = 0.059, p = 0.55 and r = -0.08, p = 0.40). HRQL was not associated with age (r = -0.048, p = 0.62) indicating acceptable discriminant validity. Regarding known groups validity the HRQL score showed the predicted pattern amongst a priori known groups regarding sex and self-perceived health. Men had higher HRQL compared to women (z = 1.958, p = 0.05). Those who rated their health as being good or very good had higher HRQL than those who rated it as fair or poor (z = -2.428, p = 0.02). There were no significant differences in HRQL score according to education and marital status. Factor analysis did not support subscales. Factor analysis of the instrument suggested a one-factor model (only one factor with eigenvalues above 1 accounting for 22% of the variance).

**Table 3:** Psychometric properties

**Psychometric property**	**Statistic**	**p value**
*Acceptability*		
% missing (summary score)	2.73%	
*Skewness*		
*Shapiro-Wilk*		*<0.05*
*Skewness test*		*0.056*
*Shapiro-Francia*		*<0.05*
*Reliability*		
Internal consistency (alpha)	0.73	
*Validity*		
Convergent (Spearman rho)		
MMSE	0.059	0.55
Disability	-0.08	0.40
Discriminant		
Age	-0.048	0.62
Known groups		
Sex (z)	1.958	0.05
Education (z)	0.751	0.45
Marital status (χ^2^)	2.989	0.39
Self perceived health (z)	-2.428	0.02
Factor analysis	1 factor solution	22% variance

### Distribution of HRQL by socio-demographic and functional variables

Tables 4 and 5 show the distribution of HRQL in the CC75C sample. HRQL in the presence of dementia does not vary by age. Men have higher HRQL than women. HRQL is higher in the 81-84 and 85-89 groups than the 90+ in men, being almost constant in women. The median HRQL score does not vary by education or type of accommodation. Married people with dementia and those that were never married have higher HRQL than their widowed counterparts. The median score does not differ much in women but it does in men, being higher in married men compared to widowers and women in general. Those with only IADL disability have better HRQL than those with no disability and those with more severe disability (including ADLs). The HRQL of those with good self-reported health is higher than those with fair/poor self-reported health. HRQL does not vary with cognition. Within those that left school before age 15, the median score is similar in those with mild and moderate severity. However, within those who left school at age 15 or later, HRQL was lower in those with moderate than those with mild dementia severity. As an additional analysis, we calculated the association between sex and severity of depression measured with the CAMDEX (the higher the score the higher the severity) using the Kruskal-Wallis equality-of-populations rank test. Men’s rank sum was 1885.5 and women’s was 4219.5 (p = 0.038).

**Table 4:** Distribution of HRQL in total and stratified by sex

**Distribution of HRQL**	**Women**	**Men**	**Total**
Age	Median & interquartile difference	Median & interquartile difference	Median & interquartile difference
81–84	14	11	12
	4	4	5
85–89	14	11	11
	5	6	5
90+	11	12	11
	8	5	3
Sex			
Men	-	-	13
			4
Women	-	-	11
			6
Education			
Left school <15 years old	13	11	12
	4	4	5
Left school ≥15 years old	13	11	12
	4	7	6
Accommodation			
House/flat	13	11	12
	4	5	5
Warden controlled	13	12	13
	7	5	5
Residential home	12	13	13
	2	6	5
Long stay hospital	3	-	3
	0	-	0
Marital status			
Married	14	10	13
	2	5	3
Widowed	12	11	11
	7	5	6
Separated/divorced	-	11	11
	-	0	0
Never married	13	13	13
	5	1	2
Disability			
No ADL or IADL	12	12	12
	4	5	5
IADL only	14	11	13
	4	2	5
ADL + IADL	12	11	11
	2	5	5
MMSE			
Moderate	13	12	12
	5	4	5
Mild	13	11	12
	4	6	5
Self-rated health			
Very good/good	13	12	13
	4	5	5
Fair/poor	11	8	9
	8	5	4

**Table 5:** Distribution of HRQL stratified by cognition

**Distribution of HRQL**	**Moderate dementia**	**Mild dementia**
Age	Median & interquartile difference	Median & interquartile difference
81–84	12	13
	5	5
85–89	12	11
	6	5
90+	12	11
	5	3
Sex		
Men	13	13
	5	4
Women	12	11
	4	6
Education		
Left school <15 years old	12	11
	6	5
Left school ≥15 years old	7	13
	9	6
Accommodation		
House/flat	11	12
	5	5
Warden controlled	13	11
	2	9
Residential home	13	11
	3	5
Long stay hospital	3	-
	0	-
Marital status		
Married	13	13
	4	3
Widowed	12	11
	6	6
Separated/divorced	11	-
	0	-
Never married	13	13
	4	3
Disability		
No ADL or IADL	10	12
	7	5
IADL only	13	12
	4	6
ADL + IADL	12	11
	6	6
Self-rated health		
Very good/good	13	13
	6	4
Fair/poor	10	8
	6	9

## Discussion

We have assessed the distribution of HRQL in very old individuals with mild to moderate dementia, a section of the population with whom it is hard to conduct this kind of research. Marital status, sex, education and self-rated health seem to be the most important variables related to HRQL. Dementia severity does not seem to be associated with HRQL in dementia.

### Strengths

There are a number of strengths in the approach we have taken. Firstly, this is a population-based sample of older old adults with dementia. The study also includes both people with dementia living in the community and institutionalised populations. The present study did not specifically set out to measure dementia-specific HRQL. However, we have been able to maximise its value by mapping most dimensions of the DEMQOL onto this project and studying the distribution of HRQL in a representative sample of the Cambridge population over 80 years old with dementia. One of the strengths of the DEMQOL conceptual framework is that it was developed using a combination of interviews with people with mild to severe dementia and their non-professional caregivers, and a review of the literature and existing instruments. The adapted version of the DEMQOL using the CC75C study questions has been shown to have acceptable psychometric properties suggesting that this scale fulfils standard criteria for acceptability, good internal consistency, and validity, namely content validity, discriminant validity and to some extent, known group differences. These properties have been tested in a population sample, providing therefore evidence of external validity of this instrument. Something no other dementia-specific measure has proved yet [18]. Another strength of our instrument is the breadth of dimensions measured compared to dimension-specific instruments such as the Progressive deteriorations Scale (PDS) [33] or a number of other dementia-specific HRQL measures [34-38].

### Weaknesses

Several limitations need to be taken into account when analysing these results. The sample used in this study was derived from several phases of a longitudinal cohort investigating HRQL in people with dementia. The use of this data may therefore result in a number of biases in the research, for example survival bias in which there might have been a selection of people with factors associated to survival or follow up such as gender, education or setting, may lead to some unrepresentative distributions of HRQL. Cognitive bias may also be affecting the results of this study given the lack of insight of people with dementia [39]. However evidence suggests that people with mild to moderate dementia are able to assess their HRQL [22,28,40,41]. There are other limitations, principally arising from the methodology of exploring HRQL, previously un-researched in this sample in which HRQL had not been assessed using existing dementia-specific measures. Firstly, since the instrument has been designed *a posteriori*, the question stems, response options and time frame of the questions were not identical to those from the DEMQOL framework. A limitation of our instrument compared to other instruments such as the ADRQL [13], QUALIDEM [20] or DEMQOL proxy [22] is that HRQL in people with severe dementia could not be analysed given the high percentage of missing values and the lack of proxy ratings for HRQL items. Compared to the original DEMQOL [22] and Bath Assessment of Subjective Quality of Life in Dementia (BASQID) [42] our instrument did not include items on worry/satisfaction with cognition and activities of daily living. Regarding the psychometric properties, some aspects such as test-retest reliability could not be evaluated due to the study design. Causality in the associations between HRQL and covariates cannot be inferred in this study, as it is cross-sectional. Finally, the distribution by ethnicity, medication or relationship with the caregiver could not be explored. These variables have been shown to be associated in previous studies [29,43].

### Summary of distribution of HRQL

Age was not associated with HRQL, a finding consistent with previous literature [42,44-47]. Findings such as the lower HRQL in men with dementia who are 90 years of age and older compared to younger groups may be due to differential mortality between gender [48]. The oldest age group is likely to have worst health than the other two age groups. The established relationship between married state and higher HRQL in men is found for men with dementia too. This could be related to social support mechanisms given one of the primary benefits of marriage for men is social connectedness [49]. According to the sex role hypothesis, this positive effect of marriage would not affect women because of the ungratifying nature of housework (usually conducted by women) and women’s primary responsibility for household chores [50,51]. This lack of effect of marriage in women and the high percentage of widowed women and married men could account for the higher HRQL of men. The finding that men have higher HRQL than women could also indicate that women had higher levels of, for example depression and anxiety, as occurs in the general population [52,53]. In fact we have found that in our sample, women have higher levels of depression than men. This finding is consistent with what is observed in clinical practice when offering support to relatives of patients with Alzheimer’s disease, and could also be related to the greater difficulties women face in terms of continuing to perform the tasks associated with their role in the family and generational context [46]. Similar results have been reported in other populations [54-56].

HRQL did not vary by levels of cognition and its association with disability is not clear. There is increasing evidence that severity of dementia may not be a major determinant of HRQL in dementia, indicating that it is possible to have good life quality at all levels of dementia severity [22,23,40,57]. This finding casts doubts on the usefulness of interventions only aimed at improving or maintaining cognition and function in order to improve the quality of life of the patient with dementia. However, there is also evidence pointing at the decreased awareness of their cognitive impairments and change of behaviour by patients with dementia [58]. This also manifests as poor awareness of deficits in ADL. In general, awareness of deficits seems to decrease with an increased severity of dementia [39,59]. This could account for the lack of association between severity and HRQL. In fact, people with moderate dementia severity who left school after age 14 had a very low HRQL score compared to those with the same level of education but higher cognition and to those who left school before that age. This may reflect that this group are more conscious of their limitations in relation to more intellectual tasks, e.g. reading for pleasure, than people with less formal education, who may be less troubled by such impairments. This could also be related with the cognitive reserve hypothesis [60]. There is evidence showing that high cognitive reserve groups have a higher cognitive decline but later [61,62]. A systematic review, found cognitive decline to be associated to HRQL [23]. Patients with high reserve and moderate dementia might be more worried about the sudden decline of their functioning compared to the other groups.

Finally, higher HRQL in those with good self-rated health compared with people with lower self-rated health has also been previously reported [47]. Whereas it is plausible that health itself may be related to HRQL, the subjective nature of the rating of both concepts and the inclusion of a similar item as part of the concept of HRQL may partly explain this association.

### Future directions

Many population-based studies on dementia exist. None of them set out to measure dementia-specific HRQL. However, a number of these studies include variables that could be used to map a dementia-specific HRQL framework into the study. Doing so would be a useful way of maximising already existing resources and allow comparison across populations and provide valuable information for policy development.

## Conclusion

To our knowledge this is the first study that has mapped an existing conceptual framework of dementia-specific HRQL onto an existing study that did not aim at measuring it, thereby describing HRQL in an under-researched section of the population of increasing clinical importance. These results have practical implications for public health policies and dementia care. They emphasise the need for taking gender into account when assessing and implementing programmes to improve HRQL. According to the present findings, improving social support and modifying sex roles that can decrease HRQL when affected by dementia such as women’s family tasks will be key in these programmes.

## Competing interests

The authors declare that they have no competing interests.

## Authors’ contributions

JP conducted the data analysis and wrote the paper. TDC, BCMS, JF, SM and JMH assisted with writing the paper. CB supervised the data analysis and development of the paper. All authors read and approved the final manuscript.

## Pre-publication history

The pre-publication history for this paper can be accessed here:

http://www.biomedcentral.com/1471-2318/14/18/prepub

## Supplementary Material

Additional file 1 Meta-analysis conducted in order to generate hypothesis that assessed validity. Click here for file
