# Supplementary material for: Health-related quality of life in the Cambridge City over-75s Cohort (CC75C): development of a dementia-specific scale and descriptive analyses
Source: BMC Geriatr. 2014 Feb 10;14:18. doi: 10.1186/1471-2318-14-18 (PMC3922243; doi:10.1186/1471-2318-14-18)
Supplement: Additional file 1 — Meta-analysis conducted in order to generate hypothesis that assessed validity. [file 1471-2318-14-18-S1.docx]

Additional file 1 Meta-analysis report

We conducted a meta-analytical review in order to generate a priori hypotheses as part as the validity assessment of the measure used. In order to identify literature, an electronic search was conducted of PsycINFO and PubMed, from inception to December 2011 using a combination of words that included quality of life and dementia as major topic Mesh terms or keywords. The criteria for inclusion were: (a) articles published in English or Spanish language on dementia-specific HRQL measures developed for use with on patients with Alzheimer's disease (AD) or mixed dementia; and (b) reporting on the association between HRQL and age, sex, education, marital status, cognition, disability and self-rated health. Exclusion criteria where generic or dimension specific scales and dementia-specific HRQL scales that only reported associations with dimensions rather than an overall score. The meta-analysis was conducted using the metan package, stata 11. Only correlations, odds ratios or mean differences between quality of life and the variables here included were used in the present analyses. These were all converted into correlations in order to conduct the meta-analysis. The assessment had to be reported by the person with dementia and not by proxy respondents. If different measures of QoL were used, only the main one was included in the analyses. If no main measure was specified, the one with a higher sample size was included. Regarding disability, if the association with both ADLs and IADLs was specified, only ADLs were included.

*Results from meta-analysis*

Results from our meta-analysis are shown below. The flowchart is shown in figure 1. The search strategy identified 848 records after duplicates were removed. Title and abstract screening eliminated 772 articles and full-text screening eliminated 47 articles. Twenty-nine studies were finally included in the meta-analysis. Figure 2 shows the Forest plots of HRQL by socio-demographic and functional variables. From these we predicted that age would not be associated with HRQL in people with dementia and that being a man, having higher cognitive function, more years of education, being married, having less disability and better self reported health would be associated with higher HRQL. A summary of these results is shown in table 1.

Figure 1. Flow chart of article selection for the meta-analysis on correlates of HRQL in people with dementia

Records identified through database searching (n=984)

Records excluded after screening (n=772)

Records screened after duplicates removed (n=848)

Studies excluded after evaluation (n=47)

Studies included in meta-analysis (n=29)

Full articles assessed for eligibility (n=76)

Additional records identified through other sources (n=0)

Figure 2. Forest plots of HRQL by socio-demographic and functional variables.

Age

References:

Chan, I. W.-P., Chu, L.-W., Lee, P. W. H., Li, S.-W., & Yu, K.-K. (2011). Effects of cognitive function and depressive mood on the quality of life in Chinese Alzheimer’s disease patients in Hong Kong. *Geriatrics & gerontology international*, *11*(1), 69–76.

Conde-Sala, J. L., Garre-Olmo, J., Turro-Garriga, O., Lopez-Pousa, S., & Vilalta-Franch, J. (2009). Factors related to perceived quality of life in patients with Alzheimer’s disease: the patient's perception compared with that of caregivers. *International journal of geriatric psychiatry*, *24*, 585–594.

Fuh, J.-L., & Wang, S.-J. (2006). Assessing quality of life in Taiwanese patients with Alzheimer’s disease. *International journal of geriatric psychiatry*, *21*(2), 103–7.

Hurt, C., Bhattacharyya, S., Burns, A., Camus, V., Liperoti, R., Marriott, A., Nobili, F., et al. (2008). Patient and caregiver perspectives of quality of life in dementia. An investigation of the relationship to behavioural and psychological symptoms in dementia. *Dementia and geriatric cognitive disorders*, *26*(2), 138–46.

Lucas-Carrasco, R., Lamping, D. L., Banerjee, S., Rejas, J., Smith, S. C., & Gómez-Benito, J. (2010). Validation of the Spanish version of the DEMQOL system. *International psychogeriatrics*, *22*(4), 589–97.

Smith, S., Lamping, D., Banerjee, S., Harwood, R., Foley, B., Smith, P., Cook, J., et al. (2005). Measurement of health-related quality of life for people with dementia: development of a new instrument (DEMQOL) and an evaluation of current methodology. *Health technology assessment (Winchester, England)*, *9*(10), 1–93.

Smith, S., Lamping, D., Banerjee, S., Harwood, R., Foley, B., Smith, P., Cook, J. C., et al. (2007). Development of a new measure of health-related quality of life for people with dementia: DEMQOL. *Psychological medicine*, *37*(5), 737–46.

Trigg, R., Skevington, S., & Jones, R. (2007). How can we best assess the quality of life of people with dementia? the Bath Assessment of Subjective Quality of Life in Dementia (BASQID). *The Gerontologist*, *47*(6), 789–97.

Zimmerman, S., Sloane, P. D., Williams, C. S., Reed, P. S., Preisser, J. S., Eckert, J. K., Boustani, M., et al. (2005). Dementia care and quality of life in assisted living and nursing homes. *The Gerontologist*, *45 Spec No*(1), 133–46.

Beer, C., Flicker, L., Horner, B., Bretland, N., Scherer, S., Lautenschlager, N. T., Schaper, F., et al. (2010). Factors associated with self and informant ratings of the quality of life of people with dementia living in care facilities: a cross sectional study. *PloS one*, *5*(12), e15621.

Sex

References:

Chan, I. W.-P., Chu, L.-W., Lee, P. W. H., Li, S.-W., & Yu, K.-K. (2011). Effects of cognitive function and depressive mood on the quality of life in Chinese Alzheimer’s disease patients in Hong Kong. *Geriatrics & gerontology international*, *11*(1), 69–76.

Conde-Sala, J. L., Garre-Olmo, J., Turro-Garriga, O., Lopez-Pousa, S., & Vilalta-Franch, J. (2009). Factors related to perceived quality of life in patients with Alzheimer’s disease: the patient's perception compared with that of caregivers. *International journal of geriatric psychiatry*, *24*, 585–594.

Zimmerman, S., Sloane, P. D., Williams, C. S., Reed, P. S., Preisser, J. S., Eckert, J. K., Boustani, M., et al. (2005). Dementia care and quality of life in assisted living and nursing homes. *The Gerontologist*, *45 Spec No*(1), 133–46.

Beer, C., Flicker, L., Horner, B., Bretland, N., Scherer, S., Lautenschlager, N. T., Schaper, F., et al. (2010). Factors associated with self and informant ratings of the quality of life of people with dementia living in care facilities: a cross sectional study. *PloS one*, *5*(12), e15621.

Lucas-Carrasco, R., Lamping, D. L., Banerjee, S., Rejas, J., Smith, S. C., & Gómez-Benito, J. (2010). Validation of the Spanish version of the DEMQOL system. *International psychogeriatrics*, *22*(4), 589–97.

Smith, S., Lamping, D., Banerjee, S., Harwood, R., Foley, B., Smith, P., Cook, J., et al. (2005). Measurement of health-related quality of life for people with dementia: development of a new instrument (DEMQOL) and an evaluation of current methodology. *Health technology assessment (Winchester, England)*, *9*(10), 1–93.

Smith, S., Lamping, D., Banerjee, S., Harwood, R., Foley, B., Smith, P., Cook, J. C., et al. (2007). Development of a new measure of health-related quality of life for people with dementia: DEMQOL. *Psychological medicine*, *37*(5), 737–46.

Education

References:

Chan, I. W.-P., Chu, L.-W., Lee, P. W. H., Li, S.-W., & Yu, K.-K. (2011). Effects of cognitive function and depressive mood on the quality of life in Chinese Alzheimer’s disease patients in Hong Kong. *Geriatrics & gerontology international*, *11*(1), 69–76.

Conde-Sala, J. L., Garre-Olmo, J., Turro-Garriga, O., Lopez-Pousa, S., & Vilalta-Franch, J. (2009). Factors related to perceived quality of life in patients with Alzheimer’s disease: the patient's perception compared with that of caregivers. *International journal of geriatric psychiatry*, *24*, 585–594.

Fuh, J.-L., & Wang, S.-J. (2006). Assessing quality of life in Taiwanese patients with Alzheimer’s disease. *International journal of geriatric psychiatry*, *21*(2), 103–7.

Lucas-Carrasco, R., Lamping, D. L., Banerjee, S., Rejas, J., Smith, S. C., & Gómez-Benito, J. (2010). Validation of the Spanish version of the DEMQOL system. *International psychogeriatrics*, *22*(4), 589–97.

Marital status

References:

Chan, I. W.-P., Chu, L.-W., Lee, P. W. H., Li, S.-W., & Yu, K.-K. (2011). Effects of cognitive function and depressive mood on the quality of life in Chinese Alzheimer’s disease patients in Hong Kong. *Geriatrics & gerontology international*, *11*(1), 69–76.

Conde-Sala, J. L., Garre-Olmo, J., Turro-Garriga, O., Lopez-Pousa, S., & Vilalta-Franch, J. (2009). Factors related to perceived quality of life in patients with Alzheimer’s disease: the patient's perception compared with that of caregivers. *International journal of geriatric psychiatry*, *24*, 585–594.

Zimmerman, S., Sloane, P. D., Williams, C. S., Reed, P. S., Preisser, J. S., Eckert, J. K., Boustani, M., et al. (2005). Dementia care and quality of life in assisted living and nursing homes. *The Gerontologist*, *45 Spec No*(1), 133–46.

Cognition

References:

Bhattacharya, S., Vogel, A., Hansen, M.-L. H., Waldorff, F. B., & Waldemar, G. (2010). Generic and disease-specific measures of quality of life in patients with mild Alzheimer’s disease. *Dementia and geriatric cognitive disorders*, *30*(4), 327–33.

Chan, I. W.-P., Chu, L.-W., Lee, P. W. H., Li, S.-W., & Yu, K.-K. (2011). Effects of cognitive function and depressive mood on the quality of life in Chinese Alzheimer’s disease patients in Hong Kong. *Geriatrics & gerontology international*, *11*(1), 69–76.

Conde-Sala, J. L., Garre-Olmo, J., Turro-Garriga, O., Lopez-Pousa, S., & Vilalta-Franch, J. (2009). Factors related to perceived quality of life in patients with Alzheimer’s disease: the patient's perception compared with that of caregivers. *International journal of geriatric psychiatry*, *24*, 585–594.

Edelman, P., Ma, B. R. F., Msw, D. K., Fulton, B. R., & Kuhn, D. (2004). Comparison of Dementia-Specific Quality of Life Measures in Adult Day Centers. *Home Health Care Services Quarterly*, *23*(1), 25–42.

Edelman, P., Fulton, B. R., Kuhn, D., & Chang, C.-H. (2005). A comparison of three methods of measuring dementia-specific quality of life: perspectives of residents, staff, and observers. *The Gerontologist*, *45 Spec No*(1), 27–36.

Fuh, J.-L., & Wang, S.-J. (2006). Assessing quality of life in Taiwanese patients with Alzheimer’s disease. *International journal of geriatric psychiatry*, *21*(2), 103–7.

Hoe, J., Katona, C., Roch, B., & Livingston, G. (2005). Use of the QOL-AD for measuring quality of life in people with severe dementia--the LASER-AD study. *Age and ageing*, *34*(2), 130–5.

Hoe, J., Katona, C., Orrell, M., & Livingston, G. (2007). Quality of life in dementia: care recipient and caregiver perceptions of quality of life in dementia: the LASER‐AD study. *International Journal of Geriatric Psychiatry*, *22*, 1031–1036.

Huang, H.-L., Chang, M. Y., Tang, J. S.-H., Chiu, Y.-C., & Weng, L.-C. (2009). Determinants of the discrepancy in patient- and caregiver-rated quality of life for persons with dementia. *Journal of clinical nursing*, *18*(22), 3107–18.

Hurt, C. S., Banerjee, S., Tunnard, C., Whitehead, D. L., Tsolaki, M., Mecocci, P., Kloszewska, I., et al. (2010). Insight, cognition and quality of life in Alzheimer’s disease. *Journal of neurology, neurosurgery, and psychiatry*, *81*(3), 331–6.

Hurt, C., Bhattacharyya, S., Burns, A., Camus, V., Liperoti, R., Marriott, A., Nobili, F., et al. (2008). Patient and caregiver perspectives of quality of life in dementia. An investigation of the relationship to behavioural and psychological symptoms in dementia. *Dementia and geriatric cognitive disorders*, *26*(2), 138–46.

León-Salas, B., Logsdon, R. G., Olazarán, J., Martínez-Martín, P., & The Msu-Adru. (2011). Psychometric properties of the Spanish QoL-AD with institutionalized dementia patients and their family caregivers in Spain. *Aging & mental health*, *15*(6), 775–83.

León-Salas, B., Olazarán, J., Muñiz, R., González-Salvador, M. T., & Martínez-Martín, P. (2011). Caregivers’ estimation of patients' quality of life (QoL) in Alzheimer's disease (AD): an approach using the ADRQL. *Archives of gerontology and geriatrics*, *53*(1), 13–8.

Logsdon, R. G., Gibbons, L. E., McCurry, S. M., & Teri, L. (2002). Assessing quality of life in older adults with cognitive impairment. *Psychosomatic medicine*, *64*(3), 510–9.

Novelli, M. M. P. C., Nitrini, R., & Caramelli, P. (2010). Validation of the Brazilian version of the quality of life scale for patients with Alzheimer’s disease and their caregivers (QOL-AD). *Aging & mental health*, *14*(5), 624–31. doi:10.1080/13607861003588840

Selai, C. E., Trimble, M. R., Rossor, M. N., & Harvey, R. J. (2001). Assessing quality of life in dementia: Preliminary psychometric testing of the Quality of Life Assessment Schedule (QOLAS). *Neuropsychological Rehabilitation*, *11*(3/4), 219–243.

Selwood, A., Thorgrimsen, L., & Orrell, M. (2005). Quality of life in dementia--a one-year follow-up study. *International journal of geriatric psychiatry*, *20*(3), 232–7.

Smith, S., Lamping, D., Banerjee, S., Harwood, R., Foley, B., Smith, P., Cook, J., et al. (2005). Measurement of health-related quality of life for people with dementia: development of a new instrument (DEMQOL) and an evaluation of current methodology. *Health technology assessment (Winchester, England)*, *9*(10), 1–93.

Thorgrimsen, L., Selwood, A., Spector, A., Royan, L., Lopez, M. de M., Woods, R. T., & Orrell, M. (2003). Whose Quality of Life Is It Anyway? *Alzheimer Disease and Associated Disorders*, *17*(4), 201–208.

Trigg, R., Skevington, S., & Jones, R. (2007). How can we best assess the quality of life of people with dementia? the Bath Assessment of Subjective Quality of Life in Dementia (BASQID). *The Gerontologist*, *47*(6), 789–97.

Trigg, R., Watts, S., Jones, R., & Tod, A. (2011). Predictors of quality of life ratings from persons with dementia: the role of insight. *International journal of geriatric psychiatry*, *26*(1), 83–91.

Vogel, A., Mortensen, E. L., Hasselbalch, S. G., Andersen, B. B., & Waldemar, G. (2006). Patient versus informant reported quality of life in the earliest phases of Alzheimer’s disease. *International journal of geriatric psychiatry*, *21*, 1132–1138.

Wolak, A., Novella, J.-L., Drame, M., Guillemin, F., Di Pollina, L., Ankri, J., Aquino, J.-P., et al. (2009). Transcultural adaptation and psychometric validation of a French-language version of the QoL-AD. *Aging & mental health*, *13*(4), 593–600.

Beer, C., Flicker, L., Horner, B., Bretland, N., Scherer, S., Lautenschlager, N. T., Schaper, F., et al. (2010). Factors associated with self and informant ratings of the quality of life of people with dementia living in care facilities: a cross sectional study. *PloS one*, *5*(12), e15621.

Lucas-Carrasco, R., Lamping, D. L., Banerjee, S., Rejas, J., Smith, S. C., & Gómez-Benito, J. (2010). Validation of the Spanish version of the DEMQOL system. *International psychogeriatrics*, *22*(4), 589–97.

Disability

References:

Bhattacharya, S., Vogel, A., Hansen, M.-L. H., Waldorff, F. B., & Waldemar, G. (2010). Generic and disease-specific measures of quality of life in patients with mild Alzheimer’s disease. *Dementia and geriatric cognitive disorders*, *30*(4), 327–33.

Conde-Sala, J. L., Garre-Olmo, J., Turro-Garriga, O., Lopez-Pousa, S., & Vilalta-Franch, J. (2009). Factors related to perceived quality of life in patients with Alzheimer’s disease: the patient's perception compared with that of caregivers. *International journal of geriatric psychiatry*, *24*, 585–594.

Edelman, P., Fulton, B. R., Kuhn, D., & Chang, C.-H. (2005). A comparison of three methods of measuring dementia-specific quality of life: perspectives of residents, staff, and observers. *The Gerontologist*, *45 Spec No*(1), 27–36.

Fuh, J.-L., & Wang, S.-J. (2006). Assessing quality of life in Taiwanese patients with Alzheimer’s disease. *International journal of geriatric psychiatry*, *21*(2), 103–7.

Hoe, J., Katona, C., Roch, B., & Livingston, G. (2005). Use of the QOL-AD for measuring quality of life in people with severe dementia--the LASER-AD study. *Age and ageing*, *34*(2), 130–5.

Hoe, J., Katona, C., Orrell, M., & Livingston, G. (2007). Quality of life in dementia: care recipient and caregiver perceptions of quality of life in dementia: the LASER‐AD study. *International Journal of Geriatric Psychiatry*, *22*, 1031–1036.

León-Salas, B., Logsdon, R. G., Olazarán, J., Martínez-Martín, P., & The Msu-Adru. (2011). Psychometric properties of the Spanish QoL-AD with institutionalized dementia patients and their family caregivers in Spain. *Aging & mental health*, *15*(6), 775–83.

León-Salas, B., Olazarán, J., Muñiz, R., González-Salvador, M. T., & Martínez-Martín, P. (2011). Caregivers’ estimation of patients' quality of life (QoL) in Alzheimer's disease (AD): an approach using the ADRQL. *Archives of gerontology and geriatrics*, *53*(1), 13–8.

Lin Kiat Yap, P., Yen Ni Goh, J., Henderson, L. M., Min Han, P., Shin Ong, K., Si Ling Kwek, S., Yi Hui Ong, E., et al. (2008). How do Chinese patients with dementia rate their own quality of life? *International psychogeriatrics*, *20*(3), 482–93.

Logsdon, R. G., Gibbons, L. E., McCurry, S. M., & Teri, L. (2002). Assessing quality of life in older adults with cognitive impairment. *Psychosomatic medicine*, *64*(3), 510–9.

Lucas-Carrasco, R., Lamping, D. L., Banerjee, S., Rejas, J., Smith, S. C., & Gómez-Benito, J. (2010). Validation of the Spanish version of the DEMQOL system. *International psychogeriatrics*, *22*(4), 589–97.

Novelli, M. M. P. C., Nitrini, R., & Caramelli, P. (2010). Validation of the Brazilian version of the quality of life scale for patients with Alzheimer’s disease and their caregivers (QOL-AD). *Aging & mental health*, *14*(5), 624–31. doi:10.1080/13607861003588840

Selai, C. E., Trimble, M. R., Rossor, M. N., & Harvey, R. J. (2001). Assessing quality of life in dementia: Preliminary psychometric testing of the Quality of Life Assessment Schedule (QOLAS). *Neuropsychological Rehabilitation*, *11*(3/4), 219–243.

Smith, S., Lamping, D., Banerjee, S., Harwood, R., Foley, B., Smith, P., Cook, J., et al. (2005). Measurement of health-related quality of life for people with dementia: development of a new instrument (DEMQOL) and an evaluation of current methodology. *Health technology assessment (Winchester, England)*, *9*(10), 1–93.

Smith, S., Lamping, D., Banerjee, S., Harwood, R., Foley, B., Smith, P., Cook, J. C., et al. (2007). Development of a new measure of health-related quality of life for people with dementia: DEMQOL. *Psychological medicine*, *37*(5), 737–46.

Snow, a L., Dani, R., Souchek, J., Sullivan, G., Ashton, C. M., & Kunik, M. E. (2005). Comorbid psychosocial symptoms and quality of life in patients with dementia. *The American journal of geriatric psychiatry*, *13*(5), 393–401.

Trigg, R., Watts, S., Jones, R., & Tod, A. (2011). Predictors of quality of life ratings from persons with dementia: the role of insight. *International journal of geriatric psychiatry*, *26*(1), 83–91.

Wolak, A., Novella, J.-L., Drame, M., Guillemin, F., Di Pollina, L., Ankri, J., Aquino, J.-P., et al. (2009). Transcultural adaptation and psychometric validation of a French-language version of the QoL-AD. *Aging & mental health*, *13*(4), 593–600.

Zimmerman, S., Sloane, P. D., Williams, C. S., Reed, P. S., Preisser, J. S., Eckert, J. K., Boustani, M., et al. (2005). Dementia care and quality of life in assisted living and nursing homes. *The Gerontologist*, *45 Spec No*(1), 133–46.

Self-rated health

Healthy vs. unhealthy (84.5 vs. 78.9) p=0.021

References:

Lucas-Carrasco, R., Lamping, D. L., Banerjee, S., Rejas, J., Smith, S. C., & Gómez-Benito, J. (2010). Validation of the Spanish version of the DEMQOL system. *International psychogeriatrics*, *22*(4), 589–97.

Table 1. Systematic review: Effect size of socio-demographic and clinical variables on HRQL in people with mild and moderate dementia

|  | Number of studies | Correlation (r) | 95% confidence interval |
| --- | --- | --- | --- |
| Age | 10 | 0.02 | -0.03, 0.08 |
| Sex | 7 | 0.09 | 0.03, 0.15 |
| Education | 4 | 0.10 | 0.01, 0.19 |
| Marital status | 3 | 0.63 | 0.58, 0.68 |
| Cognition | 25 | 0.20 | 0.17, 0.23 |
| Disability | 19 | 0.13 | -0.13, -0.09 |
| Self-reported health (means and p value) | 1 | Healthy vs. unhealthy (84.5 vs. 78.9) | p=0.021 |
